# Multispectral Sensor Calibration and Characterization for sUAS Remote Sensing

**DOI:** 10.3390/s19204453

**Published:** 2019-10-14

**Authors:** Baabak Mamaghani, Carl Salvaggio

**Affiliations:** Chester F. Carlson Center for Imaging Science, Digital Imaging and Remote Sensing Laboratory, Rochester Institute of Technology, 54 Lomb Memorial Drive, Rochester, NY 14623, USA; salvaggio@cis.rit.edu

**Keywords:** calibration, MicaSense RedEdge, spectral sensor, sensor, radiance, reflectance, error propagation

## Abstract

This paper focuses on the calibration of multispectral sensors typically used for remote sensing. These systems are often provided with “factory” radiometric calibration and vignette correction parameters. These parameters, which are assumed to be accurate when the sensor is new, may change as the camera is utilized in real-world conditions. As a result, regular calibration and characterization of any sensor should be conducted. An end-user laboratory method for computing both the vignette correction and radiometric calibration function is discussed in this paper. As an exemplar, this method for radiance computation is compared to the method provided by MicaSense for their RedEdge series of sensors. The proposed method and the method provided by MicaSense for radiance computation are applied to a variety of images captured in the laboratory using a traceable source. In addition, a complete error propagation is conducted to quantify the error produced when images are converted from digital counts to radiance. The proposed methodology was shown to produce lower errors in radiance imagery. The average percent error in radiance was −10.98%, −0.43%, 3.59%, 32.81% and −17.08% using the MicaSense provided method and their “factory” parameters, while the proposed method produced errors of 3.44%, 2.93%, 2.93%, 3.70% and 0.72% for the blue, green, red, near infrared and red edge bands, respectively. To further quantify the error in terms commonly used in remote sensing applications, the error in radiance was propagated to a reflectance error and additionally used to compute errors in two widely used parameters for assessing vegetation health, NDVI and NDRE. For the NDVI example, the ground reference was computed to be 0.899 ± 0.006, while the provided MicaSense method produced a value of 0.876 ± 0.005 and the proposed method produced a value of 0.897 ± 0.007. For NDRE, the ground reference was 0.455 ± 0.028, MicaSense method produced 0.239 ± 0.026 and the proposed method produced 0.435 ± 0.038.

## 1. Introduction

No sensor is perfect. Based on the application that is required, certain sensors are capable of producing acceptable data without any correction. On the other hand, critical applications require the highest accuracy that is achievable [1]. For this reason, it is important to regularly calibrate any sensor that is used for any kind of data collection. Even two sensors manufactured to perform the same task may produce different results under identical circumstances. Sensors can also degrade over time. Some of the changes in the sensor response can be attributed to the use and environmental conditions that the sensor is put through. These conditions include but are not limited to: temperature, storage and physical handling. In addition, the response of sensors can simply change over time as the hardware ages [2]. For these reasons, it is very important for sensors to be calibrated as often as possible.

Accuracy of data is arguably the most important part of any scientific research. For fields of study that involve imagery, the behavior of sensors is integral in the accuracy of the data [3]. While Charge-Coupled Devices (CCD) were proposed and verified in the 1970s as imaging sensors and while they used to be the most widely used imaging technology, they still had issues. Errors in CCD fabrication as well as any errors in the behavior of the electronic device contribute to the noise produced in the pixels [4]. Since then, Complementary Metal-Oxide Semiconductor (CMOS) technology has become more prominent [5]. CMOS imaging sensors also produce error in their recorded pixels values. Various work has been done to analyze and correct noise in CMOS image sensors [6,7,8]. It is important for every user to know the accuracy and expected variation of their data and how one might reduce the error that does exists [9].

This paper aims to develop a technique for characterizing and calibrating any kind of spectral sensor. Some of these sensors are loaded with previously measured metadata values which are used for calibrating any image captured by the sensor and these values are never updated. This reduces the accuracy of the final image produced by the sensor. For the purposes of this paper, the relative spectral response curves are characterized, while the vignette correction, row-gradient correction and radiometric calibration coefficients are calibrated. While the methods presented in this paper can be utilized for any spectral sensor, this study focuses on a multispectral sensor that is primarily used for agricultural remote sensing.

## 2. Background

Traditionally, remote sensing data collections were accomplished from manned aircraft and satellite based platforms. Satellite sensors were calibrated using National Institute of Standards and Technology (NIST)-traceable equipment pre-launch and were calibrated in-situ afterwards. On the other hand, manned aircraft sensors can be calibrated as often as the user desires using NIST standards. This is an advantage, because if any abnormality is detected in the results, the sensor can be investigated further in a laboratory environment. This assures the highest quality characterization and calibration. Recently, technological improvements in small unmanned aircraft systems (sUAS) have evolved to the point where they are a cost-effective solution for a variety of commercial, as well as research applications while maintaining a high level of accuracy. This has led to sUAS instrumentation being utilized more often for remote sensing purposes. Some examples of sUAS applications include but are not limited to: bridge inspections [10], cultural heritage [11], gas pipeline inspections [12] and vegetation [13,14,15]. It is very important that the imagery captured for all of those applications and many more, are calibrated properly.

In terms of calibration, sensors attached to sUAS platforms have the same advantage as manned aircraft sensors, as they can be taken off and calibrated whenever desired. All calibration methods require an accurate reference standard, which will yield reliable results when combined with a strong protocol [16]. Below are a few examples of the various methods for calibrating sensors on both satellite and unmanned aircraft platforms.

### 2.1. Satellite

Calibration of satellite sensors occurs before they are launched into orbit. However, these sensors can degrade due to mechanical and electrical issues and even exposure to ultraviolet and other radiations in the space environment [17]. On board calibration is accomplished for various Landsat sensors with the use of lamps, blackbodies, various optical components and even a neutral density filter [18]. In addition, unique techniques have been developed to use both clouds and oceans as calibration targets for satellite sensors [19,20,21] and Pseudo Invariant Calibration Sites (PICS) have been used in recent years for calibration purposes [22,23,24]. A nice overview of satellite instrumentation calibration was written by Chander et al. [25], which included but was not limited to: Simultaneous Nadir Overpasses (SNO), vicarious ground based calibrations, PICS, clouds, Rayleigh scattering and even the sun, moon and stars.

### 2.2. Unmanned Aircraft Systems (UAS)

As mentioned, the advantage of calibrating a UAS sensor is the ability to perform calibration using NIST-traceable equipment in a laboratory. Below are a few examples from previous studies. Berni et al. calibrated both a multispectral and a thermal sensor. Their multispectral sensor radiance calibration utilized a uniform light source along with an integrating sphere, while their thermal camera was calibrated with a blackbody source and the MODTRAN radiative transfer code [14]. Sheng et al. also calibrated their thermal camera by imaging a surface over a range of known temperatures [26]. Vicarious calibration has been utilized by both Pozo et al. and Li et al. [27,28]. Pozo’s technique measured artificial targets and established a relationship between the targets and the radiance of the surfaces, while Li’s technique applied the same technique to calibrated tarps and calculated the top-of-atmosphere (TOA) radiances to compute calibration coefficients. Hakala et al. performed laboratory calibration of their hyperspectral camera by using both a monochromator and a source-based reference panel configuration. The monochromator was utilized for spectral calibration while a reference panel configuration was used for absolute radiance calibration [29]. Aasen et al. performs a dark current calibration, a vignette correction and a radiometric response correction [30]. Kelcey et al. calibrated imagery by noise reduction, dark offset, vignette correction by flat fielding and corrects lens distortion using Agisoft Lens software [31]. Vignette correction is the process of correcting the fall off of digital count intensity at the edges of the image frame.

### 2.3. MicaSense RedEdge

Designed to assist agricultural professionals with crop management, the MicaSense RedEdge (MicaSense, Seattle, WA, USA) is a five band multispectral sensor. The sensor captures 8 cm ground sample distance (GSD) at 122 m, up to 1 capture per second with a 47.2° field of view (FOV), for all five bands. Furthermore, the sensor comes equipped with a downwelling light sensor (DLS) which measures the incident light, providing a hemispherical measurement above the detector surface. The bands of the RedEdge camera and their respective characteristics are listed in Table 1.

MicaSense’s calibration procedure utilizes various metadata parameters that accompany each image produced by each individual RedEdge sensor [32]. This raises concerns for two main reasons. First, these metadata parameters are fixed for the lifetime of the sensor unless a new “factory” calibration is performed. Second, some of the early RedEdge-3 sensors produced were not radiometric calibrated individually. Instead, they were loaded with default radiometric and vignette correction terms. While these default values could produce good results, they will not produce the most accurate results. Their process for converting raw digital count imagery into radiance, $L_{i}\left(x,y\right)$ [W/m${}^{2}$/sr/nm], for band *i*, is described below in Equations (1)–(4) [32].

(1) $$L_{i}\left(x,y\right)=V_{i}\left(x,y\right)\frac{a_{1,i}}{g_{i}}\frac{\rho_{i}\left(x,y\right)-\rho_{BL,i}}{t_{e,i}+a_{2,i}y-a_{3,i}t_{e,i}y}$$

where $V_{i}\left(x,y\right)$ is the vignette correction function map, $a_{1,i}$, $a_{2,i}$ and $a_{3,i}$ are radiometric calibration coefficients, $\rho_{i}\left(x,y\right)$ is the raw digital count image, $\rho_{BL,i}$ is the dark level, $g_{i}$ is the gain, $t_{e,i}$ is the exposure time [s] and *y* is the pixel row number. The vignette map, $V_{i}\left(x,y\right)$, is represented as

(2) $$V_{i}\left(x,y\right)=\frac{1}{k_{i}}$$

where $k_{i}$ is a correction factor

(3) $$k_{i}=1+k_{0,i}r_{i}+k_{1,i}r_{i}^{2}+k_{2,i}r_{i}^{3}+k_{3,i}r_{i}^{4}+k_{4,i}r_{i}^{5}+k_{5,i}r_{i}^{6}$$

where $k_{0,i}$ through $k_{5,i}$ are polynomial correction coefficients and $r_{i}$ is the distance of the pixel to the vignette centers

(4) $$r_{i}=\sqrt{{\left(x-c_{x,i}\right)}^{2}+{\left(y-c_{y,i}\right)}^{2}}$$

where $c_{x,i}$ and $c_{y,i}$ represent the vignette center and *i* denotes the spectral band number. Now, by combining all the above Equations (1)–(4), the radiance image, $L_{i}\left(x,y\right)$ can be computed.

## 3. Methods

### 3.1. Proposed Radiometric Calibration Methodology

While the RedEdge sensor provides a method to convert images from digital count into radiance [32], a separately proposed method is presented here. This proposed methodology allows users to calibrate their sensors whenever it is deemed necessary. This will ensure that the sensor is converting newly captured digital count imagery into radiance imagery as accurately as possible. In addition, use of this proposed methodology allows for the production of a fully parameterized radiance error image. The proposed calibration process is a two step process: first, determine the vignette and row gradient correction, and second, compute a spectral radiometric calibration coefficient.

#### 3.1.1. Vignette and Row-Gradient Correction

As stated before, vignette correction refers to correcting the fall off of digital count intensity at the edges of an image, while row gradient correction corrects for a fall off of digital count intensity from top to the bottom of the image. To calculate both the vignette and row gradient correction factors simultaneously, the sensor needs to be presented with a spatially static (flat) radiance field. In order to do this, the sensor should be aimed directly into the exit port of a integrating sphere, where an image is captured and then normalized, by dividing out the gain and exposure time. It should be noted that the exit aperture of the integrating sphere should be larger than the field of view (FOV) of the sensor. This ensures that the images captured by the sensor are fully illuminated with the spatially uniform and stable radiance from the sphere. From these images, the combined vignette and row-gradient factor correction surface, $V_{i}\left(x,y,T,t_{e,i},g_{i}\right)$, is derived as

(5) $$V_{i}\left(x,y,T,t_{e,i},g_{i}\right)=\frac{\rho_{sphere,i}\left(x,y\right)-\rho_{BL,i}\left(T,t_{e,i},g_{i}\right)}{\rho_{sphere,i}\left(c_{x,i},c_{y,i}\right)-\rho_{BL,i}\left(T,t_{e,i},g_{i}\right)}$$

where $\rho_{sphere,i}\left(x,y\right)$ is the normalized integrating sphere image, $\rho_{BL,i}\left(T,t_{e,i},g_{i}\right)$ is the dark level bias (Section 3.3), $\rho_{sphere,i}\left(c_{x,i},c_{y,i}\right)$ is the normalized pixel value at the vignette center $c_{x,i},c_{y,i}$ and *i* denotes the spectral band number. This allows for any image to be corrected of optical fall-off, sensor imperfections and dark-level bias using the following equation

(6) $$\rho_{c,i}\left(x,y\right)=\frac{\rho_{i}\left(x,y\right)-\rho_{BL,i}\left(T,t_{e,i},g_{i}\right)}{V_{i}\left(x,y,T,t_{e,i},g_{i}\right)}$$

where, $\rho_{c,i}\left(x,y\right)$ is the vignette and row-gradient corrected image. Now that the images can be corrected for fall-off, radiometric calibration can take place. Ultimately, the calibration equaiton is dependent on the light-level, gain, integration time and even temperature.

#### 3.1.2. Calibration Coefficient Computation

To convert a raw digital count image to a radiance image, the following equation is utilized for each of the camera’s spectral bands, *i*

(7) $$L_{i}\left(x,y\right)=a_{i}\frac{\rho_{c,i}\left(x,y\right)}{g_{i}t_{e,i}}$$

where $L_{i}\left(x,y\right)$ is the radiance imagery, $a_{i}$ is the radiometric calibration coefficient, $\rho_{c,i}\left(x,y\right)$ is the vignette corrected image, $g_{i}$ is the gain and $t_{e,i}$ is the exposure time [s].

To compute the band specific calibration coefficient, $a_{i}$, the sensor needs to be shown a series of *S* varying brightness level radiance spectra spanning the operational range that the camera will be used under, namely $\left[L_{1},L_{2},L_{3},...,L_{S}\right]$. These radiance levels are provided using a traceable source. By using the relative response functions, band-effective radiance, $L_{s,i}$, can be computed by

(8) $$L_{s,i}=\frac{\int_{\lambda }L_{s}\left(\lambda \right)RSR_{i}\left(\lambda \right)d\lambda }{\int_{\lambda }\left(\lambda \right)RSR_{i}\left(\lambda \right)d\lambda }$$

where $RSR_{i}\left(\lambda \right)$ is the relative spectral response curve for band *i* (Section 3.2). $a_{i}$ can be derived through a no-intercept least-squares regression as:

(9) $$a_{i}={\left(X_{i}^{t}X_{i}\right)}^{-1}X_{i}^{t}Y_{i}$$

where

$$X_{i}=\left[\begin{array}{c} \frac{1}{g_{i,1}t_{e,i,1}}{\bar{\rho }}_{c,1,i}\\ \frac{1}{g_{i,2}t_{e,i,2}}{\bar{\rho }}_{c,2,i}\\ \frac{1}{g_{i,3}t_{e,i,3}}{\bar{\rho }}_{c,3,i}\\ \vdots \\ \frac{1}{g_{i,S}t_{e,i,S}}{\bar{\rho }}_{c,S,i} \end{array}\right]$$

(10) $${\bar{\rho }}_{c,s,i}=\sum_{x}\sum_{y}\frac{\rho_{c,s,i}\left(x,y\right)}{N_{x}M_{y}}$$

where $N_{x}$ and $M_{y}$ are the number of pixels in the column and rows of the image, respectively and

$$Y_{i}=\left[\begin{array}{c} L_{1,i}\\ L_{2,i}\\ L_{3,i}\\ \vdots \\ L_{S,i} \end{array}\right]$$

### 3.2. Relative Spectral Response

**Figure 1:**
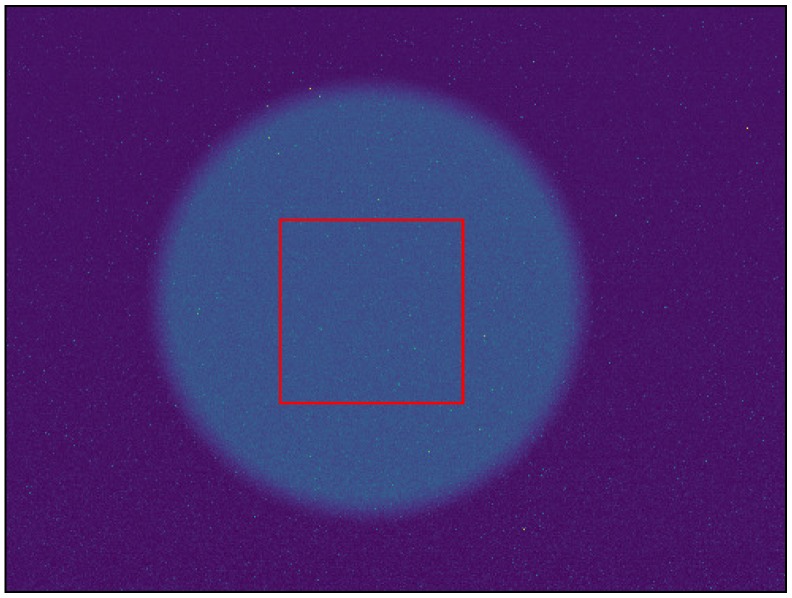
Example region of interest (red rectangle) from relative spectral response data set. Pixels from center of sphere (exit port of mini integrating sphere) were averaged for every image captured. This was accomplished for all 250 images captured (400 nm–900 nm, 2 nm step size) for all bands.

**Figure 2:**
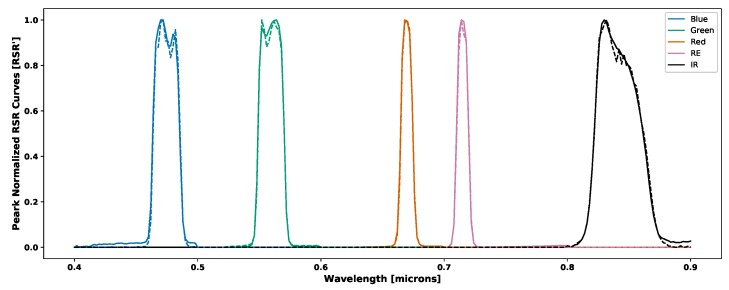
Peak normalized relative spectral response of RedEdge-3 (solid lines) and RedEdge-M (dashed lines) sensors. Small variations can be seen in all channels of both sensors. RedEdge-3 contains small divots in the blue and green channels, while the Red, RE and NIR channels have small but noticeable shifts between the two sensors.

RSR curves were computed using a method similar to the one described by Mamaghani et al. [33]. The RSR determination methodology is shown in Equations (11)–(13).

(11) $$DC_{norm_{i}}\left(\lambda \right)=\frac{{\bar{DC}}_{i}\left(\lambda \right)}{g_{i}t_{e_{i}}}$$

(12) $$RSR_{i}\left(\lambda \right)=\frac{0.9975(DC_{norm_{i}}\left(\lambda \right)-b)}{\Phi (\lambda )}$$

(13) $${\hat{RSR}}_{i}\left(\lambda \right)=\frac{RSR_{i}\left(\lambda \right)}{max(RSR_{i}\left(\lambda \right))}$$

where ${\bar{DC}}_{i}\left(\lambda \right)$ is the average digital count over a region of interest (an example of which can be seen in Figure 1), $g_{i}$ is the gain, $t_{e_{i}}$ is the exposure time [s], *b* is a shift factor that is equal to the lowest non-zero value of $DC_{norm_{i}}\left(\lambda \right)$, Φ(λ) [W] is the spectral radiant flux, $RSR_{i}\left(\lambda \right)$ is the relative spectral response, ${\hat{RSR}}_{i}\left(\lambda \right)$ is the peak normalized relative spectral response and *i* denotes the spectral band number. The peak normalized relative spectral response functions for the two RedEdge sensors used in this study can be seen in Figure 2.

The variations that are seen between the RedEdge-3 and RedEdge-M relative spectral response curves (Figure 2) is a good demonstration of the importance for calibration. These are two sensors produced by the same company with the same specifications, yet they produce slightly different responses. These variations may be attributed to slight variations in their manufacture or perhaps aging and exposure of the filter materials to environmental factors.

### 3.3. Dark Current

To characterize the dark current of the RedEdge sensors, light was blocked from the lenses by physically covering the entrance aperture and placing the sensor in a light tight bag (a photographer’s film changing bag). The sensor was then placed into three different environments: cold (2.78 °C), ambient (25.56 °C) and hot (37.22 °C). A series of 30 images were captured at every gain and manual integration time/exposure setting. With four gain settings (1×, 2×, 4× and 8×) along with 21 exposure settings (ranging 0.0066 ms–24.5 ms). For a single combination (temperature, gain and exposure), the pixels in all 30 images were averaged to estimate the spectral dark current. In addition to the MicaSense RedEdge camera, a HOBO TidbiT MX Temp 400 temperature logger (Onset, Bourne, MA, USA) was placed inside the light tight bag to record the temperature every 10 s throughout the experiment. This was done to ensure the temperature did not radically change throughout the experiment period.

### 3.4. Comparison Metrics

Images from the integrating sphere were captured and converted to radiance using both the MicaSense RedEdge provided methodology and the proposed method described above. The output from the sphere was measured using a calibrated spectroradiometer (a Spectra Vista Corporation HR-1024i field portable spectroradiometer with a 4 degree foreoptic as well as an Analytic Spectral Devices FieldSpec 3 Spectroradiometer with a 3 degree foreoptic) as reference against which to compare the derived spectral radiance images. Various light levels, gains and exposure combinations were used in this evaluation. Light levels were selected based on the simulating the highest radiance seen on Earth and multiplying it by the average Earth reflectance (18%).

### 3.5. Error Propagation

Given a calibrated radiance from the above approach, it is of keen interest to characterize the expected error on this derived value. An error propagation was conducted to assess the accuracy of the radiance imagery produced. Error propagation is calculated with the general form

(14) R=F(X,Y,...)

(15) $$\delta R=\sqrt{{\left(\frac{\partial F}{\partial X}\delta X\right)}^{2}+{\left(\frac{\partial F}{\partial Y}\delta Y\right)}^{2}\;+\;...}$$

where *R* is a function of *X* and *Y* and δR is the standard error in *R*.

Using Equation (15) and applying it to Equations (7) and (1), the error propagation for every pixel in the radiance imagery can be computed for the methods utilized in this study.

#### 3.5.1. MicaSense Method Error

(16) $$\begin{array}{cc} \delta L_{i}\left(x,y\right) & =[{\left(\frac{\partial L_{i}\left(x,y\right)}{\partial g_{i}}\delta g_{i}\right)}^{2}+{\left(\frac{\partial L_{i}\left(x,y\right)}{\partial p_{i}\left(x,y\right)}\delta p_{i}\left(x,y\right)\right)}^{2}+{\left(\frac{\partial L_{i}\left(x,y\right)}{\partial t_{e,i}}\delta t_{e,i}\right)}^{2}+\\ & {\left(\frac{\partial L_{i}\left(x,y\right)}{\partial V_{i}\left(x,y\right)}\delta V_{i}\left(x,y\right)\right)}^{2}+{\left(\frac{\partial L_{i}\left(x,y\right)}{\partial a_{1,i}}\delta a_{1,i}\right)}^{2}+{\left(\frac{\partial L_{i}\left(x,y\right)}{\partial a_{2,i}}\delta a_{2,i}\right)}^{2}+{\left(\frac{\partial L_{i}\left(x,y\right)}{\partial a_{3,i}}\delta a_{3,i}\right)}^{2}{]}^{1/2} \end{array}$$

where

(17) $$\frac{\partial L_{i}\left(x,y\right)}{\partial g_{i}}=-V_{i}\left(x,y\right)\frac{a_{1,i}}{g_{i}^{2}}\frac{p_{i}\left(x,y\right)-p_{BL,i}}{t_{e,i}+a_{2,i}y-a_{3,i}t_{e,i}y}$$

(18) $$\frac{\partial L_{i}\left(x,y\right)}{\partial p_{i}\left(x,y\right)}=V_{i}\left(x,y\right)\frac{a_{1,i}}{g_{i}}\frac{1}{t_{e,i}+a_{2,i}y-a_{3,i}t_{e,i}y}$$

(19) $$\frac{\partial L_{i}\left(x,y\right)}{\partial t_{e,i}}=-V_{i}\left(x,y\right)\frac{a_{1,i}}{g_{i}}\frac{\left(p_{i}\left(x,y\right)-p_{BL,i}\right)\left(1-a_{3,i}y\right)}{{\left(t_{e,i}+a_{2,i}y-a_{3,i}t_{e,i}y\right)}^{2}}$$

(20) $$\frac{\partial L_{i}\left(x,y\right)}{\partial V_{i}\left(x,y\right)}=\frac{a_{1,i}}{g_{i}}\frac{\rho_{i}\left(x,y\right)-\rho_{BL,i}}{t_{e,i}+a_{2,i}y-a_{3,i}t_{e,i}y}$$

(21) $$\frac{\partial L_{i}\left(x,y\right)}{\partial a_{1,i}}=V_{i}\left(x,y\right)\frac{1}{g_{i}}\frac{\rho_{i}\left(x,y\right)-\rho_{BL,i}}{t_{e,i}+a_{2,i}y-a_{3,i}t_{e,i}y}$$

(22) $$\frac{\partial L_{i}\left(x,y\right)}{\partial a_{2,i}}=-V_{i}\left(x,y\right)\frac{a_{1,i}}{g_{i}}\frac{y(\rho_{i}\left(x,y\right)-\rho_{BL,i})}{{\left(t_{e,i}+a_{2,i}y-a_{3,i}t_{e,i}y\right)}^{2}}$$

(23) $$\frac{\partial L_{i}\left(x,y\right)}{\partial a_{3,i}}=V_{i}\left(x,y\right)\frac{a_{1,i}}{g_{i}}\frac{yt_{e,i}\left(\rho_{i}\left(x,y\right)-\rho_{BL,i}\right)}{{\left(t_{e,i}+a_{2,i}y-a_{3,i}t_{e,i}y\right)}^{2}}$$

where $\delta g_{i}$ is the standard error in gain, $\delta t_{e,i}$ is the standard error in exposure time, $\delta \rho_{i}\left(x,y\right)$ is the standard error in the raw image, $\delta V_{i}\left(x,y\right)$ is the standard error of the vignette correction, & $\delta a_{1,i}$, $\delta a_{2,i}$ and $\delta a_{3,i}$ are the standard errors of the radiometric calibration coefficients. The general standard error form is given in Equation (24), while the standard error for regression estimates is shown by Equation (26).

(24) $$\delta =\frac{\sigma_{pop}}{\sqrt{N_{samp}}}$$

where δ is the standard error, $\sigma_{pop}$ is the standard deviation of the population and $N_{samp}$ is the number of samples. While the MicaSense Radiance Error (Equation (16)) contains seven different components, not all of these variables could be computed. When the radiance error is computed for the MicaSense methodology, only gain, exposure time and image error is considered. This is because the RedEdge multispectral sensors are provided with “factory” values for the radiometric calibration and vignette correction coefficients. Therefore, there is no way to compute the individual error contributions and the standard error will be set to 0 for the following variables: $V_{i}\left(x,y\right)$, $a_{1,i}$, $a_{2,i}$ and $a_{3,i}$.

#### 3.5.2. Proposed Method Error

(25) $$\begin{array}{cc} \delta L_{i}\left(x,y\right) & ={\left[{\left(\frac{\partial L_{i}\left(x,y\right)}{\partial a_{i}}\delta a_{i}\right)}^{2}+{\left(\frac{\partial L_{i}\left(x,y\right)}{\partial p_{c,i}}\delta p_{c,i}\right)}^{2}+{\left(\frac{\partial L_{i}\left(x,y\right)}{\partial g_{i}}\delta g_{i}\right)}^{2}+{\left(\frac{\partial L_{i}\left(x,y\right)}{\partial t_{e,i}}\delta t_{e,i}\right)}^{2}\right]}^{1/2}\\ \; & ={\left[{\left(\frac{p_{c,i}}{g_{i}t_{e,i}}\delta a_{i}\right)}^{2}+{\left(\frac{a_{i}}{g_{i}t_{e,i}}\delta p_{c,i}\right)}^{2}+{\left(\frac{-a_{i}p_{c,i}}{g_{i}^{2}t_{e,i}}\delta g_{i}\right)}^{2}+{\left(\frac{-a_{i}p_{c,i}}{g_{i}t_{e,i}^{2}}\delta t_{e,i}\right)}^{2}\right]}^{1/2}\\ \; & ={\left[\frac{p_{c,i}^{2}{\left(\delta a_{i}\right)}^{2}+a_{i}^{2}{\left(\delta p_{c,i}\right)}^{2}}{g_{i}^{2}t_{e,i}^{2}}+\frac{a_{i}^{2}p_{c,i}^{2}\left[t_{e,i}^{2}{\left(\delta g_{i}\right)}^{2}+g_{i}^{2}{\left(\delta t_{e,i}\right)}^{2}\right)]}{g_{i}^{4}t_{e,i}^{4}}\right]}^{1/2} \end{array}$$

where $\delta p_{c,i}$ is the standard error of the vignette corrected image and δa is the standard error of the radiometric calibration coefficient. Because *a* was computed using a least squares regression the standard error computation for this variable changes. Below is the standard error for a regression estimate.

(26) $$\delta_{est}=\frac{\sqrt{\frac{\sum_{i=1}^{N_{samp}}\left(Y_{i}-Y^{\prime }\right)}{N_{samp}-2}}}{\sqrt{\sum_{i=1}^{N_{samp}}\left(X_{i}-\bar{X}\right)}}$$

where $\delta_{est}$ is the standard error of the estimate, *Y* is the dependent variable value for observation and $Y^{\prime }$ is the predicted dependent variable value, $X_{i}$ is the independent variable value for observation and $\bar{X}$ is the mean of the independent variables.

#### 3.5.3. Reflectance Error

This study also investigates the error in reflectance imagery. While the Empirical Line Method (ELM) is the most well known method to convert digital count/radiance imagery into reflectance, other techniques have been employed in recent years. A recent example is utilizing the At-Altitude Radiance Ratio (AARR), which was implemented with promising results using the MicaSense RedEdge sensor. AARR produces a reflectance image by dividing each spectral band radiance image by the corresponding band-effective downwelling radiance. Equation (27) demonstrates this process [33].

(27) $$\rho_{i}=\frac{L_{s,i}}{DLS_{i}}$$

(28) $$DLS_{i}=\frac{E_{solar,i}^{{}^{\prime }}}{\pi }cos\left(\sigma^{\prime }\right)\tau_{i}^{{}^{\prime }}+L_{↓solar,i}$$

where $DLS_{i}$ is the downwelling light sensor radiance recorded by the MicaSense RedEdge, $\rho_{i}$ is the reflectance factor, $L_{s,i}$ is the band effective spectral radiance, $E_{solar,i}^{{}^{\prime }}$ is the spectral exoatmospheric solar irradiance, $\sigma^{\prime }$ is the solar zenith angle, $\tau_{i}^{{}^{\prime }}$ is the spectral transmission from space to the sUAS, $L_{↓solar,i}$ is the solar scattered downwelling sky radiance propagating on to the sUAS and *i* denotes the spectral band number.

The error in reflectance is shown below in Equation (29)

(29) $$\begin{array}{cc} \delta \rho_{i} & =\sqrt{{\left(\frac{\partial \rho_{i}}{\partial L_{s,i}}\delta L_{s,i}\right)}^{2}+{\left(\frac{\partial \rho_{i}}{\partial DLS_{i}}\delta DLS_{i}\right)}^{2}}\\ \; & =\sqrt{{\left(\frac{1}{DLS_{i}}\delta L_{s,i}\right)}^{2}+{\left(\frac{-L_{s,i}}{DLS_{i}^{2}}\delta DLS_{i}\right)}^{2}}\\ \; & =\sqrt{\frac{1}{DLS_{i}^{2}}\left(\delta L_{s,i}^{2}+\frac{L_{s,i}^{2}}{DLS_{i}^{2}}\delta DLS_{i}^{2}\right)} \end{array}$$

## 4. Data Collection

For the purposes of data collection, a NIST-traceable HELIOS Uniform integrating sphere (Labsphere, Sutton, NH, USA, SN:0720165129) along with two Plasma External Lamp (PEL) sources, an OL Series 750 Automated Spectroradiometric Measurement System (Optronic Laboratories, Orlando, FL, USA, SN:05613133), a Newport Hand-Held Optical Meter Model 1918-R (Newport, Irvine, CA, USA, SN: 17160), a SVC HR-1024i field portable spectroradiometer (Spectra Vista Corporation, Poughkeepsie, NY, USA), an Analytic Spectral Devices FieldSpec 3 Spectroradiometer (Analytik Ltd, Cambridge, UK), a MicaSense RedEdge-3 multispectral sensor (SN:1713165), a MicaSense RedEdge-M (SN:RM01-1806106-SC) and a DJI Matrice 100 quadcopter (SZ DJI Technology Co., Nanshan District, Shenzhen, China) were used for this study. The OL Series 750 and the Newport Optical Meter were used to generate the relative spectral response curves of the MicaSense RedEdge, while the integrating sphere was used for vignette correction, row-gradient correction and radiometric calibration. The SVC and ASD spectroradiometers were used to measure the output of the integrating sphere as calibrated standards. The DJI quadcopter was used to fly the RedEdge sensor around various scenes in Western, NY. While the images captured from these scenes were utilized for other research studies, a simple case study was accomplished using a single sUAS image captured with the RedEdge-3. This case study demonstrates the effects of calibration on both radiance and reflectance sUAS imagery.

### 4.1. Dark Current

**Figure 3:**
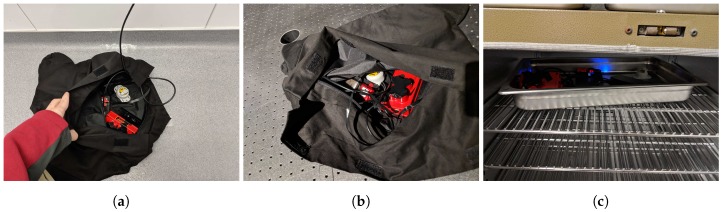
MicaSense RedEdge-3 and HOBO TidbiT MX Temp 400 during dark current testing in (**a**) cold room, (**b**) room temperature and (**c**) bench oven.

As previously stated, three various environments were used to test the dark current: a walk-in cold room (set to 2.78 °C), an ambient laboratory (25.56 °C) and a bench oven (set to 37.22 °C). Figure 3 shows the MicaSense RedEdge-3 and HOBO TidbiT MX Temp 400 in these various locations for testing. The timing of these experiments varied between the RedEdge-3 and RedEdge-M. The RedEdge-3 was tested first and the gains and exposure times were changed manually in-between image captures. This added significant down time in data collection and caused each temperature setting test to last between 1.5 and 2 h. Afterwards, it was learned the RedEdge sensor has an Application Program Interface (API) and the sensor can be controlled pragmatically. Therefore, a small script was written up that programatically changed the gains and exposure times after all 30 images were captured for the previous combination. This reduced the testing time to around a single hour.

### 4.2. Relative Spectral Response

**Figure 4:**
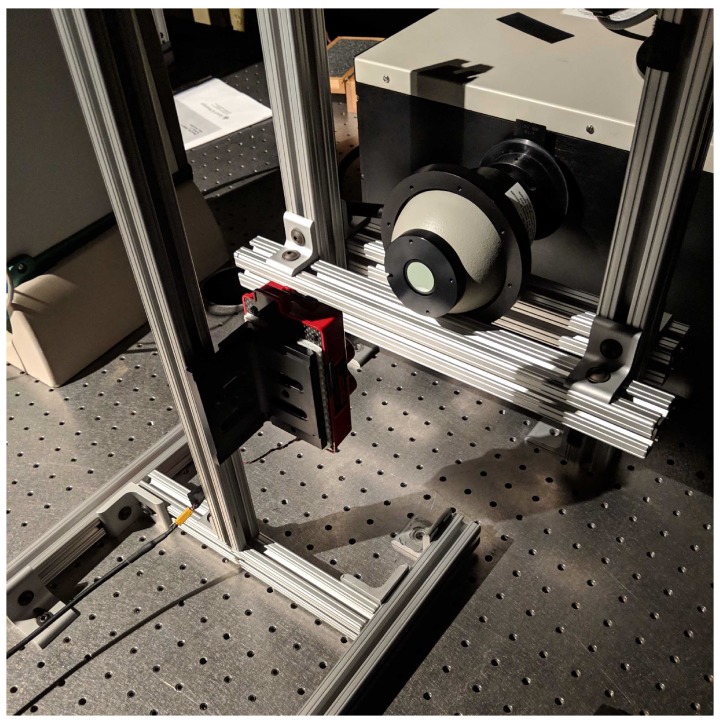
Relative spectral response curve data capture setup. Monochromator exit port connected to mini integrating sphere. RedEdge-3’s center lens is aligned with exit port of mini sphere. RedEdge-3 is attached to a custom made rig which allowed for both lateral and vertical movement.

To measure the Relative Spectral Response (RSR) curves, an OL Series 750 monochromator was utilized with 0.5 mm slits at both the entrance and exit apertures to produce a 2 nm half-bandwidth (HBW). A mini integrating sphere was attached at the exit port of the monochromator to produce uniform light, while the RedEdge was placed 11 cm away from the sphere. This produced the image seen in Figure 1. The RedEdge was situated close to the exit port of the mini integrating sphere because of the small exit port and the lower power being put into the monochromator. With a larger sphere and more input power, the sensor could be placed further back. Figure 4 depicts the monochromator configuration used. Power levels and spectral imagery were recorded at 2 nm increments from 400 nm to 900 nm.

### 4.3. Radiometric Calibration

**Figure 5:**
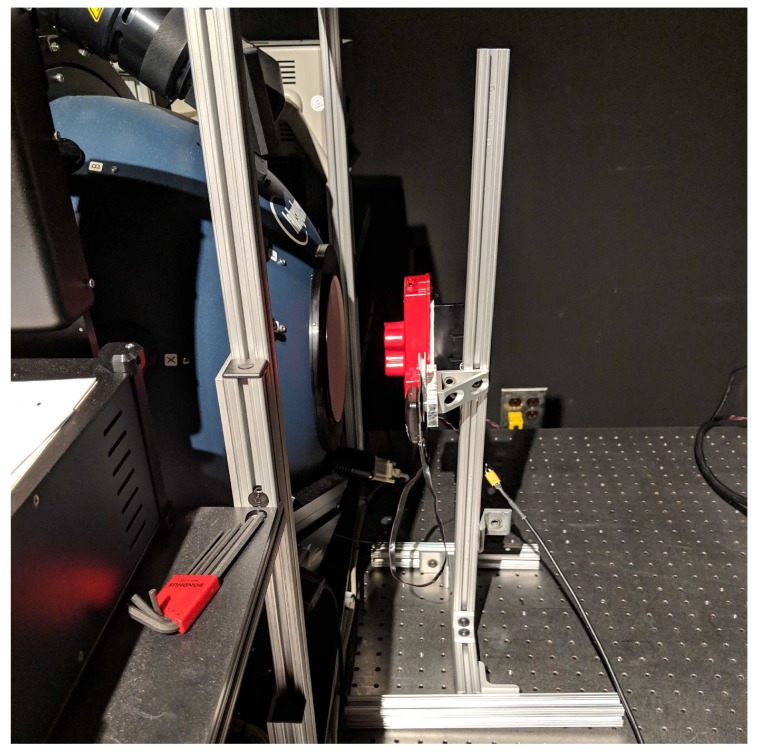
Radiometric calibration data capture setup. RedEdge-3 sensor is placed in front of the exit port of the integrating sphere. Sensor is placed far enough away to avoid stray light issues but close enough for all channels to be completely filled. RedEdge is attached to a custom made rig which allowed for both lateral and vertical movement.

**Figure 6:**
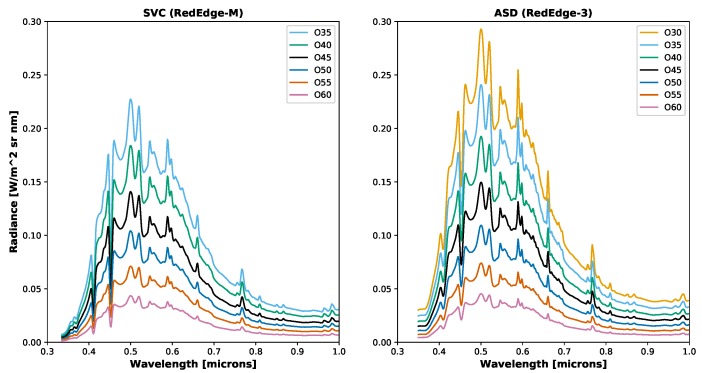
Example integrating sphere radiances. (**Left**) SVC radiances collected and used for calibrating the RedEdge-M sensor. (**Right**) ASD radiances collected and used for calibrating the RedEdge-3 sensor. As expected, the measured spectra decreased in magnitude as the PEL’s aperture was closed.

To calculate the radiometric calibration coefficient, the sensor needs to be shown *N* number of spectral radiance curves, which for the purposes of this study, *N* was selected to be seven. The setup used with the integrating sphere and Micasense RedEdge is displayed in Figure 5. The highest light level selected for the integrating sphere was computed by simulating the radiance seen on Earth and multiplying it by the average Earth reflectance (18%). This radiance is close to the radiance level produced from the integrating sphere when a single PEL source is on and is 30% closed. One of the PEL sources used in this study has a variable aperture. This is beneficial because it allows for setting the required *N* spectral radiance curves. It was chosen to use seven light levels: One lamp on, 30% (O30), 35% (O35), 40% (O40), 45% (O45), 50% (O50), 55% (O55) and 60% (O60) closed, which respectively are: 0.2928, 0.2407, 0.1924, 0.1495, 0.1093, 0.0739, 0.0453 W/m${}^{2}$/sr at their peak wavelengths (500 nm). The radiance spectra collected at these light levels can be seen in Figure 6. After the light levels were selected, all MicaSense sUAS flights conducted by the sUAS Lab at the Chester F. Carlson Center for Imaging Science at Rochester Institute of Technology were analyzed to see what gain and exposure combinations were selected when the camera was collecting field data in auto exposure mode. These 40 flights were conducted between August 2017 and March 2019, a majority of which were flown around Western, NY, USA during late spring and late fall. These flights were flown on sunny days, between 46 m and 122 m above ground level and between 10:00 and 14:00 EST to ensure that the sun was above of the scene. While all four gain levels were utilized, only a small subset of the available exposure times were utilized (0.5 ms–2.5 ms). With this knowledge in mind, the radiometric calibration data set was constrained to seven light levels, four gains and six exposure settings. Even with these constraints, which produced 168 combinations, not all combinations could be utilized for the radiometric calibration coefficient computation. Many of these combinations produced data that was either near the noise floor or near saturation. Because of this, an additional constraint was added to only use combinations in which all 5 spectral bands produced an an image in which the top 5% of pixels fell in between the noise floor and saturation.

### 4.4. Independent Radiance Test Data

Independently collected radiance test data were captured using the same experimental setup under which the original radiometric calibration imagery was acquired. Various light levels, gains and exposure combinations were captured using the RedEdge-3 and RedEdge-M cameras. All these images were converted to radiance using both the MicaSense provided approach as well as the proposed methodology. Errors were computed by comparing the average of the radiance imagery to the band integrated radiance that was captured by the SVC/ASD spectroradiometers that were observing the integrating sphere during data collection.

### 4.5. Error

To compute the overall radiance error, three separate experiments were executed in order to compute the standard deviation for each of the three primary components: gain, exposure time and image count. The error in gain and image count was computed utilizing data collected from the integrating sphere, while the error in exposure time was computed with an oscilloscope.

Various light level and exposure combinations were captured with the RedEdge sensor for the gain and image count error calculation. These combinations were selected because the light levels and exposures were able to be held constant while the gain was increased over three base 2 orders of magnitude without producing a saturated signal. This ultimately produced five combinations where gain error was computed and seven combinations for computing image error. Each of these combinations were already known to produce usable vignettes in all bands of the sensor, which allowed all the images to be corrected and averaged. As stated before, usable vignettes were determined to be viable if the top 5% of the pixel values were within the noise floor and saturation in all 5 spectral bands. Ultimately, the average pixel values (the average pixel value of an image is computed by Equation (10)) across 30 corrected images was computed for each combination and used to compute gain and digital count errors.

**Figure 7:**
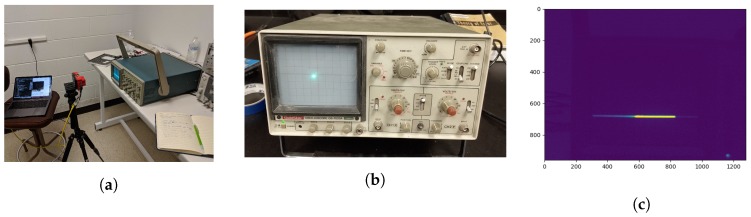
Exposure time error (**a**) setup, (**b**) example oscilloscope dot and (**c**) example RedEdge image. Images were used for analysis if the full line was imaged in the center of the frame. This ensures the proper exposure time could be measured.

Exposure time error was computed using an oscilloscope. The RedEdge sensor was setup to capture images of the oscilloscope screen as the scope scanned across the screen. The dot that scanned over the screen became recorded as a line. The length of this line was directly proportional to the exposure time of the image. Three exposure times were tested (0.5, 1 and 2.5 ms). The setup and example images can be seen below in Figure 7.

The DLS error was measured using the integrating sphere by placing the DLS in line with the exit port of the integrating sphere. 30 measurements were captured at eight different light levels and standard errors were computed for all light levels and all 5 bands.

Standard error for all of these components (gain, digital count, exposure time and DLS) is computed using Equation (24).

## 5. Results

### 5.1. Dark Current

**Figure 8:**
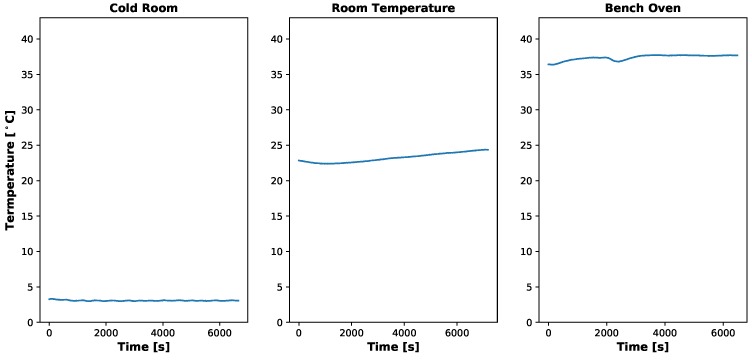
Temporal temperature profile during MicaSense RedEdge-3 dark current testing. Temperature inside the light tight bag did not change significantly during the course of the test. Data collection took about 2 h for each temperature environment because the gain and exposure time had to be changed manually in-between image captures.

**Figure 9:**
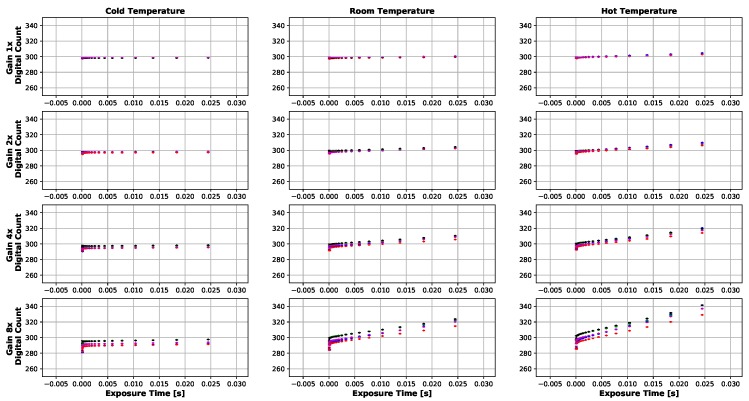
Average dark current (12-bit) produced by MicaSense RedEdge-3 sensor at various gains and exposure times. Each dot represents an average of at least 30 images. Bars are one standard deviation. Digital count values hold around 300 except for higher exposure times, gains and temperatures. The highest digital counts values seen were around 340 (24.5 ms, 8× gain and 37.22 ${}^{\circ }$C).

**Figure 10:**
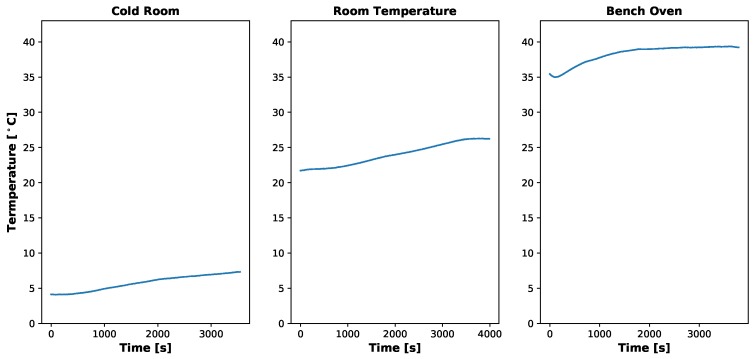
Temporal temperature profile during MicaSense RedEdge-M dark current testing. Temperature inside light tight bag did rise as test progressed but no significant changes in the digital counts were noticed. Data collection took about an hour for each temperature setting because the gain and exposure time were altered programatically using the RedEdge Application Program Interface (API).

**Figure 11:**
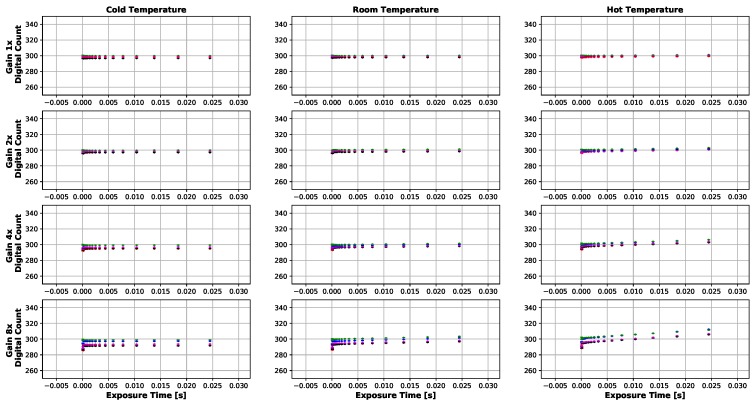
Average dark current (12-bit) produced by MicaSense RedEdge-M sensor at various gains and exposure times. Each dot represents an average of at least 30 images. Bars are one standard deviation. Digital count values hold around 300 except for higher exposure times, gains and temperatures. The highest digital counts values seen were around 320 (24.5 ms, 8× gain and 37.22 ${}^{\circ }$C). It should also be noted that the digital count values held steady regardless of the increasing temperature throughout the test.

Dark current results can be seen in Figure 8, Figure 9, Figure 10 and Figure 11. Figure 8 and Figure 10 show the temperatures of the environments the MicaSense RedEdge-3 and RedEdge-M sensors were in during the data capture, while Figure 9 and Figure 11 display the average 12-bit noise computed at each gain, exposure time and temperature. As the temperature, gain and exposure time all increased, the noise increased as well. But it should be noted, that at the normal operating range of this sensor (25 °C–26.67 °C and exposure times 0.5 ms–2.5 ms), the dark current was around 300 digital counts.

### 5.2. Radiometric Calibration

By using Equation (8), band integrated radiance values were computed for every sphere setting used. These values are displayed in Table 2.

Of all the calibration imagery collected, 25 combinations were accepted. As stated before, these combinations were determined to be viable because the top 5% of the raw pixel values were within the noise floor and saturation in all 5 spectral bands. Table 3 displays the computed radiometric calibration coefficients for both the MicaSense RedEdge-3 and RedEdge-M that were utilized in this study.

**Figure 12:**
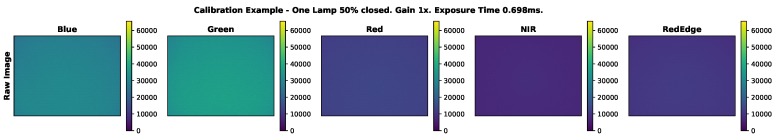
Example raw RedEdge imagery taken of the integrating sphere during calibration testing.

**Figure 13:**
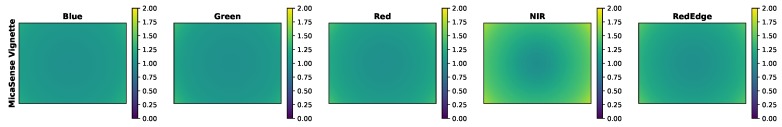
MicaSense vignette imagery. These vignettes are programmed into the particular MicaSense RedEdge-3 sensor used in this study and will never change throughout the lifetime of the sensor.

**Figure 14:**
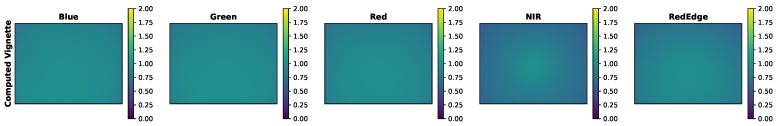
Example proposed vignettes. These vignettes are computed with the methodology proposed in this paper, for the particular light level, gain and exposure time combination (One Lamp 50% closed, Gain 2× and 0.698 ms).

**Figure 15:**
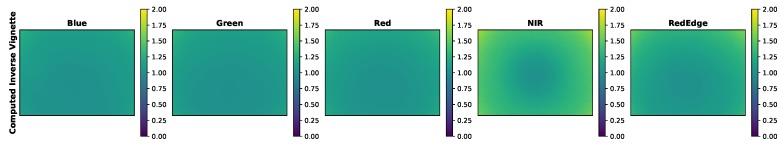
Inverse of example proposed vignettes. Proposed methodology divides the image by the vignette, while MicaSense method multiples. These images were produced to showcase the similarity between MicaSense and proposed vignettes.

**Figure 16:**
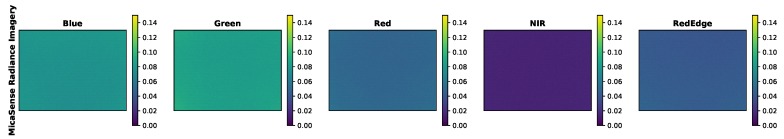
Example integrating sphere imagery converted into radiance using MicaSense radiance methodology. 3-D images are shown below for clarity.

**Figure 17:**
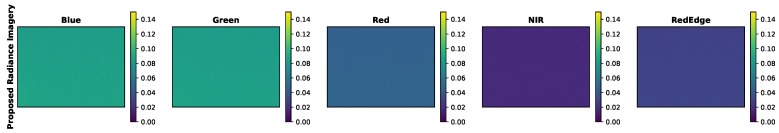
Example integrating sphere imagery converted into radiance using proposed technique. 3-D images are shown below for clarity.

**Figure 18:**
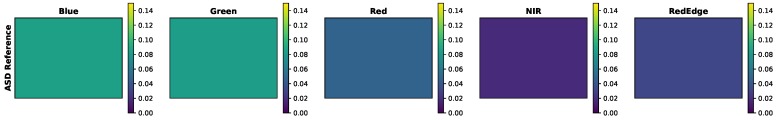
ASD reference values measured of the integrating sphere during calibration testing. RedEdge-3 RSR curves applied to ASD spectra to compute band integrated radiances, which are being displayed as images.

Both sensors produced very similar coefficients for the visible bands (Blue, Green and Red) but different coefficients were computed in the NIR and RedEdge. No significant changes were reported by MicaSense in the NIR or RedEdge bands for the RedEdge-M, which means that either the calibration run by MicaSense was performed incorrectly for those two channels or the RedEdge-3 and RedEdge-M really have different calibration coefficients. After computing these radiometric calibration coefficients with the proposed method, both digital count to radiance methods were compared. Over 160 test images were captured and converted to radiance and the percent error between the average radiance pixel (Equation (10)) and the band integrated radiance values in Table 2 were computed. An example can be seen in Figure 12, Figure 13, Figure 14, Figure 15, Figure 16, Figure 17 and Figure 18. All of these images are computed from the same image set that was captured under the following combination: One lamp on, 50% closed, gain 1×, exposure time 0.698 ms.

Figure 12 displays the raw imagery captured by the RedEdge, Figure 13 displays the vignette correction images produced using the MicaSense provided approach and metadata parameters, Figure 14 displays the proposed method’s vignette corrections surfaces and Figure 15 displays the inverse of the proposed method’s vignette corrections. The inverse of the proposed vignettes is displayed to match the multiplicative approach to this correction used by MicaSense.

The overall results are shown below. Figure 16 is the radiance imagery produced using the MicaSense provided approach and “factory” parameters while Figure 17 is the radiance imagery produced using the proposed method. Figure 18 is the band integrated sphere radiance measured with the ASD displayed as 2D images for comparison purposes.

**Figure 19:**
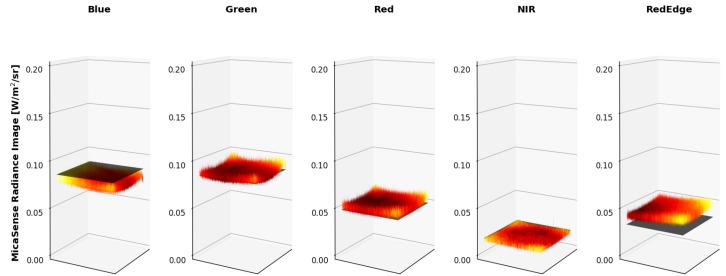
Example MicaSense radiance imagery shown as 3-D images. ASD reference is shown as a black 2-D image. Imperfections of MicaSense methodology is noticeable in the magnitude (radiance calibration coefficients) and in the edges (vignette correction) of the radiance images.

**Figure 20:**
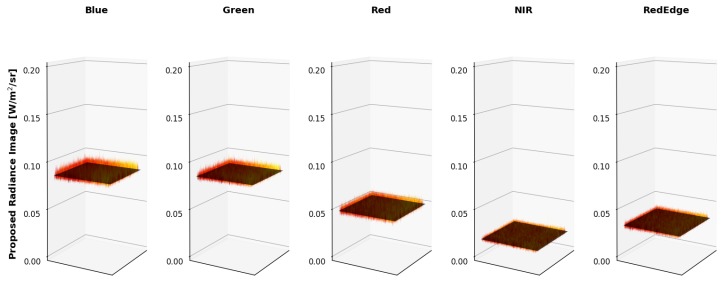
Example proposed radiance imagery shown as 3-D images. ASD reference is shown as a black 2-D image. Using the proposed methodology, the produced radiance imagery magnitudes line up with the ASD reference and the applied vignette correction flattened the images.

Since it is difficult to visualize the variation in the radiance imagery, 3 dimensional (3-D) plots were created to showcase the results of both methods (MicaSense radiance imagery in Figure 19 and proposed radiance imagery in Figure 20). The colored vignettes are the computed radiance imagery, while the black 2-D images are the ASD references. There are a few issues with the radiance imagery produced by MicaSense. The first is the magnitudes of the radiances. They do not align with the ASD reference values, most notably in the Blue and RedEdge channels. This means that the radiometric calibration coefficients are not very accurate in those bands. Furthermore, the produced radiance imagery still had some vignette features. The edges of the radiance imagery fold up, meaning the vignette correction that was utilized was also not entirely accurate. However, the radiance imagery produced by the proposed method align on top of the band integrated ASD measurements and the vignette and row-gradient correction flattened the images.

**Figure 21:**
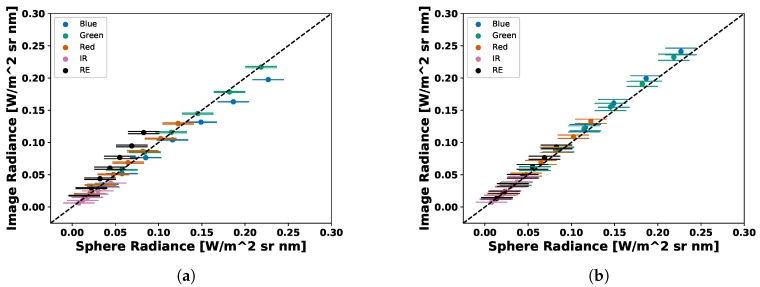
Scatter plot of average RedEdge-3 image radiance vs sphere radiance. Images converted to radiance using (**a**) MicaSense methodology (**b**) Average Vignette Proposed methodology. Proposed methodology produces less error as less spread is seen in the scatter plot.

**Figure 22:**
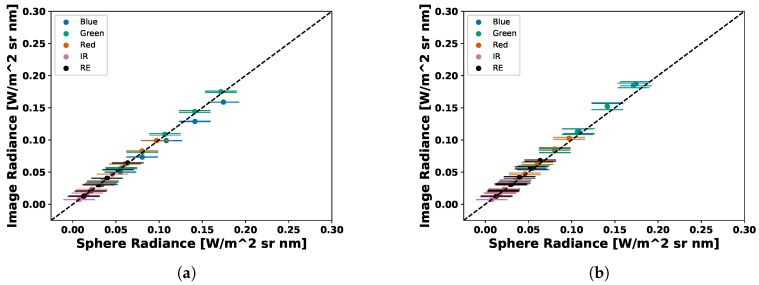
Scatter plot of average RedEdge-M image radiance vs sphere radiance. Images converted to radiance using (**a**) MicaSense methodology (**b**) Average Vignette Proposed methodology. Proposed methodology produces less error as less spread is seen in the scatter plot.

Once all test images were converted to radiance, the results were compared. Table 4 and Table 5 present the overall percent errors in radiance for both methods and both sensors. Two variations of vignettes were utilized to calibrate the test data. As stated before, to compute the radiometric calibration coefficient, $a_{i}$, 25 light level, gain and exposure combinations were accepted (top 5% of raw pixels between noise floor and saturation), which produced 25 vignettes. When calibrating test imagery, the images were corrected with: (1) averaging all 25 vignettes and (2) the closest vignette in terms of light level, gain and exposure time. After these results were calculated, scatter plots were produced to display the difference between the computed image radiance levels and the band integrated sphere radiance values. These are shown in Figure 21 and Figure 22.

Some of these radiometric calibration results stand out. All bands, except green, produced lower average percent errors when the proposed methodology was utilized as opposed to the MicaSense provided method and “factory” parameters. It is also worth noting that the RedEdge and NIR channel produced very high percent errors in RedEdge-3 radiance imagery. This is noteworthy because the RedEdge sensor was developed for agricultural applications which primarily investigate the spectral behavior in wavelengths above 700 nm. Another noticeable result is the increased error produced with the RedEdge-3 sensor across all bands, except green. This higher error could be a result of the extensive use of the RedEdge-3 over the RedEdge-M in both the calibration lab as well as in the field. The RedEdge-3 used in this study was purchased in June 2017 while the RedEdge-M was purchased in March 2018. It is also possibly that this error is a result of the RedEdge-3 not being individually calibrated. In other words, generic calibration coefficients for both the radiometric values and the vignette were built into the RedEdge-3.

**Figure 23:**
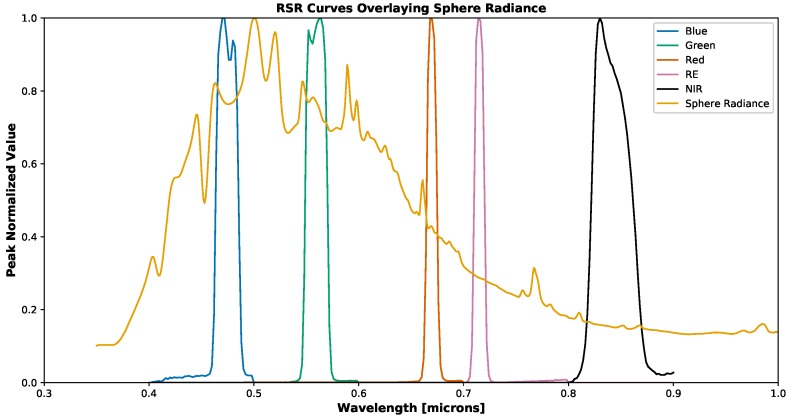
Peak normalized MicaSense RedEdge-3 RSR curves overlaid onto normalized spectral radiance curve.

Finally, the percent errors in radiance imagery were not zero for the proposed technique because of potential errors in the relative spectral response curves. As can be seen in Figure 2, there are variations with the curves. The RedEdge-3 blue RSR curve trails off all the way to 400 nm which definitely plays a result in the band integrated value. This trail off in the blue channel could also be present because not enough noise was suppressed when processing the data. Another issue is any potential shift in the RSR curves which is most noticeable in the RedEdge curve between both the 3 and M series sensors. Even a 2–3 nm shift in the rise and fall of the RSR can impact the band integrated values. To demonstrate this difference, the RSR curves of the RedEdge-3 sensor were overlaid onto a peak normalized spectral radiance curve from the integrating sphere. This overlap can be seen below in Figure 23. As shown, the band integrated radiances are: 0.1491, 0.1453, 0.0817, 0.0551 and 0.0298 W/m${}^{2}$/sr/nm for Blue, Green, Red, RedEdge and Near Infrared respectively. If a 3 nm shift to the right is applied to all bands, the resulting band integrated radiance values become: 0.1494 (+0.20%), 0.1429 (−1.65%), 0.0795 (−2.69%), 0.0543 (−1.45%), 0.0296 (−0.67%).

### 5.3. Reflectance Error

The radiance errors also carry through to reflectance (an illumination invariant space). The errors in reflectance of the RedEdge and NIR bands will be more noticeable because the radiance error in those bands are highest. This is important because two of the most commonly used metrics for vegetation health: NDVI (Normalized Difference Vegetation Index) and NDRE (Normalized Difference RedEdge) utilize these two bands [34,35]. Computation of both of these metrics is shown below in Equations (30) and (31).

(30) $$NDVI=\frac{\rho_{NIR}-\rho_{Red}}{\rho_{NIR}+\rho_{Red}}$$

(31) $$NDRE=\frac{\rho_{NIR}-\rho_{RE}}{\rho_{NIR}+\rho_{RE}}$$

**Figure 24:**
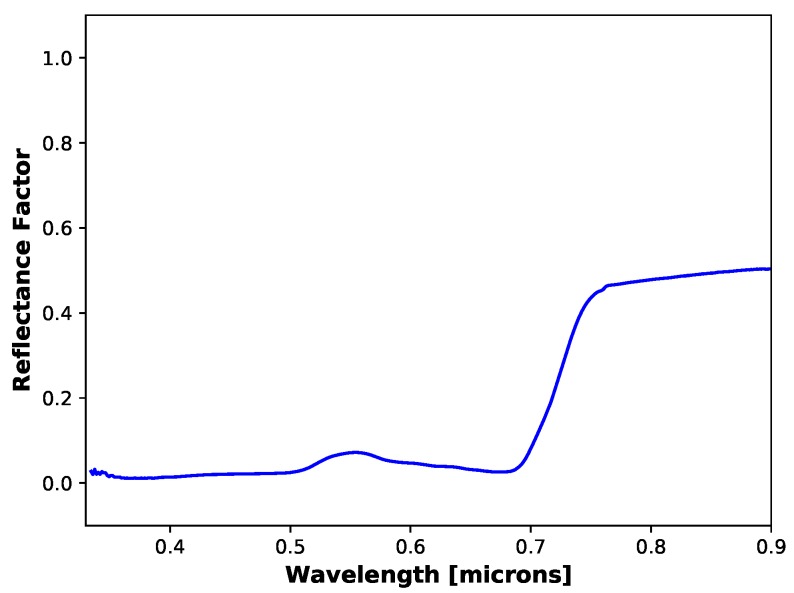
Ground reference reflectance of grass target measured with SVC during a field collection in 2018. This measurement is a single measurement of the grass target.

Below, a basic example of the resulting errors in reflectance for a grass target is shown. Figure 24 shows a ground reference reflectance spectra measured from a grass target that was collected using the SVC on a field collection in 2018. This target was measured roughly once per 45 min (at the start of every sUAS flight). When collecting the target’s reflectance spectra, a measurement of a >99% diffuse reflector, Spectralon (Labsphere, Sutton, NH, USA), was made before as a reference. Proper collection of spectra in the field consists of keeping the SVC at chest height and shoulders parallel to the sun. This ensures that the collector’s shadow is not over the target being measured. Assuming this spectra is correct, then Table 6 showcases the resulting errors of the calibration methods for a grass target as well as the computed NDVI and NDRE metrics. As can be seen, the RedEdge-3 sensor produces significantly different results for the NDRE metric.

As expected, the RedEdge-M sensor produced similar results for both NDVI and NDRE regardless of the method utilized. However, the computed NDVI and NDRE values of the RedEdge-3 sensor showed a different story. Compared to the NDVI reference of 0.899, the MicaSense method produced an NDVI of 0.876 while the proposed method produced a closer value of 0.897. This larger NDVI error produced from the MicaSense method is due to the larger radiance error of the NIR band. On the other hand, when the NDRE is computed, the MicaSense error is significantly higher because it includes both the NIR and RedEdge band which had a −17% and 32% error in the radiance domain respectively. By calibrating the RedEdge-3, the NDRE error dropped from −46% to −2.2%. If a farmer or an agricultural scientist use a sensor that has not been calibrated, their results may lead them to believe that the crop field has issues (NDRE of 0.239). This would cause them to spend time and resources to ensure the crop fields are healthy. On the other hand, a calibrated sensor would have displayed a healthy field (NDRE of 0.435) and saved both time and capital.

### 5.4. Radiance Errors

Below are the standard errors for images, gains, exposure times and downwelling light sensor. The computation of these errors was explained in Section 3.5. For digital count, gain and the DLS, errors were computed for various combinations of light level and other appropriate parameters. Below are the following tables: digital count error (Table 7), gain error (Table 8), exposure time error (Table 9), radiometric calibration coefficient error (Table 10) and DLS error (Table 11).

The DLS error was computed using both PEL sources, because the DLS is designed to measure solar irradiance, which is higher in value than the radiance being recorded by the camera sensors.

Ultimately, the errors for an example radiance image are produced in Figure 19 and Figure 20.

**Figure 25:**
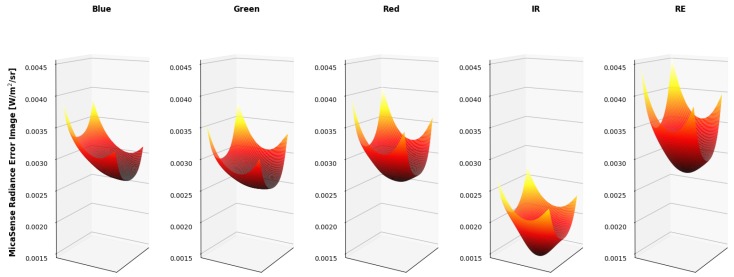
Example error of MicaSense radiance imagery. Only gain, exposure time and image error components are present in these errors. Noticeable vignette shape is seen in all channels.

**Figure 26:**
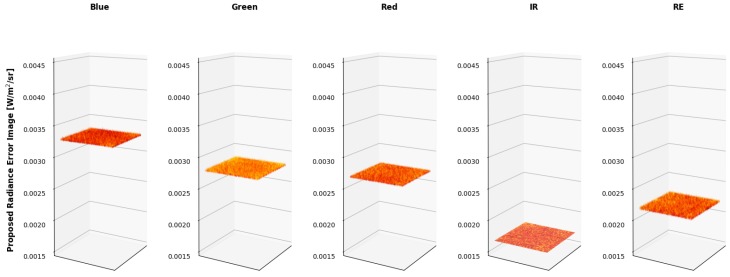
Error of proposed radiance imagery. Gain, exposure time, image and radiometric calibration coefficient error components are present. Error images are all flat for all channels, as the radiance images were.

Table 12 showcases the error in radiance as a percentage of the actual radiance image. In other words, the average pixel of the radiance error images (Figure 25 and Figure 26) were divided by the average pixel of the radiance images (Figure 19 and Figure 20).

While the radiance error images produced by both methods were close to the same magnitude, their computation was imbalanced. The proposed radiance error images included standard errors of the radiometric calibration coefficient, which included the vignette corrections. While the MicaSense methodology for radiance image computation includes radiometric calibration coefficients and a vignette correction, their standard errors were not used in the error computation. As stated before, this is because MicaSense provides constants in the metadata that are potentially unique (depends on the RedEdge sensor version) to that sensor. Therefore, the MicaSense error radiance image, Figure 25 and the percentage errors reported in Table 12 are under estimations of the actual error for the MicaSense methodology.

#### Example Reflectance Error

**Figure 27:**
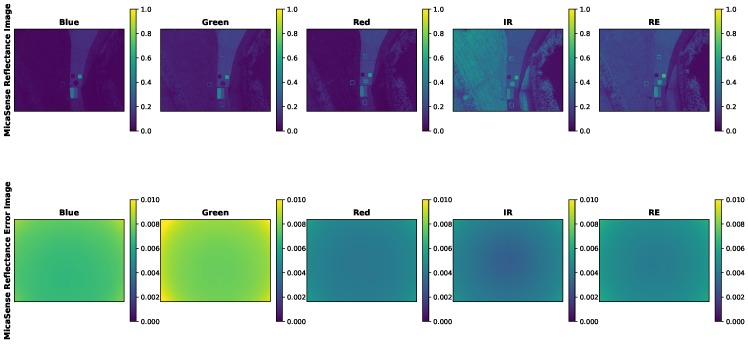
Example sUAS reflectance factor image and reflectance factor error image. Radiance image computed using MicaSense methodology and AARR used to compute reflectance factor. Reflectance factor error only contains gain, exposure time, image and DLS error. Error image is overwhelmed by the vignette shape. Reflectance factor is a unit-less quantity.

**Figure 28:**
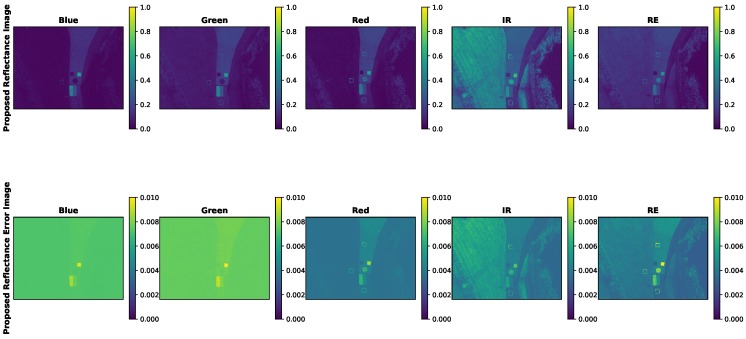
Example sUAS reflectance factor image and reflectance factor error image. Radiance image computed using proposed methodology and AARR used to compute reflectance factor. Reflectance factor error contains gain, exposure time, image, radiometric calibration coefficient and dls error. Error image contains same features as the reflectance factor image (concrete path, grass, demarcated targets and reflectance conversion panels). Reflectance factor is a unit-less quantity.

An example image from an sUAS field collect was converted to radiance using both methodologies (MicaSense and proposed) and then converted into reflectance using the AARR technique. The resulting reflectance images and reflectance errors are displayed below in Figure 27 and Figure 28.

**Figure 29:**
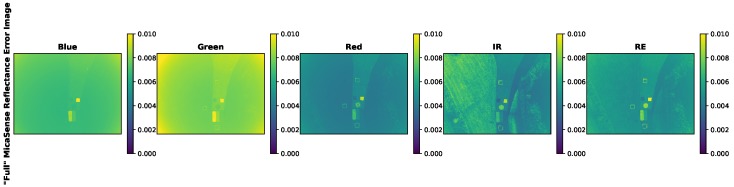
Example sUAS reflectance error image. Error image contains the measured gain, exposure time, image and dls error. While the vignette and radiometric calibration coefficient standard errors were set to 1% of their values. These error images produce the same features as the reflectance image and the proposed methodology reflectance error images. Reflectance factor is a unit-less quantity.

While the proposed methodology reflectance error images display the context of the scene, the MicaSense reflectance error images do not. This is probably due to the exclusion of the standard errors for the vignette and radiometric calibration coefficients ($V_{i}\left(x,y\right)$, $a_{1,i}$, $a_{2,i}$ and $a_{3,i}$). For this reason, the MicaSense reflectance errors for this example sUAS image were recomputed by including standard errors for the original excluded variables. Because these errors could not be computed by data collection, the radiometric calibration standard errors of the proposed method were analyzed and seen to be around 1% (except for the RedEdge band) of the actual values. Therefore, the standard errors of the vignette and radiometric calibration coefficient were set to 1% for this final example. The image below displays the the “full” MicaSense reflectance error image (Figure 29).

With the inclusion of all the potential sources of error, the MicaSense reflectance error image now looks similar to the proposed methodology. The scene is well defined with the features of the calibration panels and the grass. In addition, the MicaSense vignette’s impact can be seen in the corners of the reflectance error image.

Finally, the NDVI and NDRE of the demarcated grass were computed from both sets of reflectance imagery in which the ground reference NDVI and NDRE were 0.899 and 0.445 respectively. The MicaSense reflectance image produced an NDVI of 0.891 and an NDRE of 0.371 while the proposed method produced values of 0.905 and 0.471 respectively. As stated before, these imperfections in the MicaSense calibration methodology could cause a farmer or an agricultural professional to believe that the crops are unhealthy and need to be treated. This would cause them to waste their time and capital. Moreover, if these spectral sensors are being utilized on sUAS for bridge and/or gas pipeline inspections, it is very important to ensure that the imagery being captured is calibrated correctly. Calibrated imagery could be the difference between detecting a structural flaw on the bridge and a leak in the gas pipeline or not, which could potentially put lives at risk.

While the method proposed in this paper was tested on a MicaSense RedEdge sensor, the process outlined in this study can be utilized for any other imaging sensor. As demonstrated above, the sensor needs to have its dark current measured, relative spectral response functions computed and a number of uniform radiance levels at various gain and exposure combinations imaged, for all bands. This will allow any imaging sensor user to compute accurate radiometric calibration for their sensor, regardless of the number of spectral bands.

## 6. Future Work

### 6.1. Improving Results

To further improve the results of the proposed radiometric calibration, more ASD and SVC reference spectra could have been collected from the sphere. By collecting and averaging more spectra, the error that is present in both the ASD and SVC can be reduced. Only 25 ASD measurements and five SVC measurements were collected at each light level. These numbers were selected because of the time constraint of data collection. The integrating sphere’s output was able to be held constant (Stable Mode) for 20 min before returning to its normal state (Rest Mode). Data was collected in Stable Mode to ensure that all images captured in that time frame were recording the same radiance output. If the sphere’s stable mode could be lengthened to allow for more ASD or SVC measurements, this would provide users with a more accurate representation of the radiance that is outputted by the sphere.

In addition, the relative spectral response curves could be recollected using better equipment and methods. As seen in Figure 2, the blue channel for the RedEdge-3 sensor trails off all the way to 400 nm. This is not physically possible as the blue filter in the sensor peaks at 475 nm and has a FWHM of 20 nm. Also, a single image was captured at every wavelength sample for collection of the RSR curves in order to save time. In order to produce the most accurate RSR curves, each channel should have been aligned with the exit aperture of the monochromator and analyzed separately. In addition, the sampling interval could have been improved by dropping from 2 nm to 1 nm and more than one image could have been captured at each wavelength interval. This would have provided a more sampled version of the RSR curves and multiple images at each wavelength would have allowed for averaging to reduce any noise produced by the RedEdge sensor.

Finally, if more radiance levels are analyzed, this could produce a more accurate radiometric calibration coefficient. Only seven light levels (O30, O35, O40, O45, O50, O55 and O60) were tested. Again, this was done to save time during data collection. A finer sampling interval could have been used (2.5% closure instead of 5% per level). This would have been 13 different light levels used for analysis, which would have formed a rigorous radiometric calibration coefficient.

### 6.2. Further Calibration Study

In order to determine how often sensors need to be calibrated, frequent calibration should be performed over the course of a few months during a collection season. This would also allow users to understand how utilization of their sensors in the field can potentially degrade the quality of the data collected. The authors recommend that radiometric calibration be performed every month over the course of a collection season. If the radiometric calibration coefficients, *a* and the combined vignette and row-gradient factor correction surface, $V_{i}\left(x,y,T,t_{e,i},g_{i}\right)$, significantly change between months, then weekly calibration should be performed. However, if no noticeable difference is detected, then calibration should be performed every few months.

## 7. Conclusions

Ultimately, this paper proposed a methodology for characterizing and calibrating any spectral sensor. The proposed methodology includes characterizing the relative spectral response curves, measuring the dark current level, computing a vignette and row-gradient correction and computing radiometric calibration coefficients. These techniques were tested on a MicaSense RedEdge-3 and RedEdge-M, two multispectral sensors that come preloaded with constant metadata values for calibrating digital count imagery into radiance. Overall, the tests showed that the proposed technique produced lower errors in radiance imagery than the MicaSense methodology for the RedEdge-3 sensor. While both the relative spectral response curves and the dark current measurements performed in this study matched well with the reported values by MicaSense, the vignette and radiometric calibration coefficients did not. The measured spectral curves aligned at the reported center wavelengths and produced very similar FWHM values and the average dark current level that was measured produced comparable results to the metadata level. The difference in the overall radiance imagery results came from the vignette and radiometric calibration coefficients. It was noticed that the vignette produced by the sensor change based on light level, gain and exposure time, while the MicaSense methodology utilized only a single set of vignettes. While the standard deviations in the radiance error are higher in the proposed methodology, this is because of the low number (25) of combinations utilized in the computation of the radiometric calibration coefficient, $a_{i}$ and the combined vignette and row-gradient factor correction surface, $V_{i}\left(x,y,T,t_{e,i},g_{i}\right)$. The key takeaways are the average percent error in radiance imagery, which were: −10.98%, −0.43%, 3.59%, 32.81% and −17.08% using the MicaSense method and 3.44%, 2.93%, 2.93%, 3.70% and 0.72% using the proposed method for the Blue, Green, Red, RedEdge and Near Infrared bands respectively. Overall, this study has demonstrated the importance in both characterizing and calibrating sensors and has shown how any error can affect results in both the radiance and reflectance domain. 

## Figures and Tables

**Table 1:** MicaSense RedEdge spectral bands with respective center wavelengths and bandwidth values.

Band Name	Wavelengths [nm]	FWHM [nm]
Blue	475	20
Green	560	20
Red	668	10
Red Edge	717	10
Near IR	840	40

**Table 2:** Band integrated sphere radiances. Values were computed by applying RedEdge-3 RSR curves to the measured sphere radiances. All spectral band columns have units of W/m${}^{2}$/sr/nm.

% Closed	Peak Radiance [W/m${}^{2}$/sr/nm]	Blue	Green	Red	NIR	RedEdge
30	0.2928	0.2268	0.2186	0.1226	0.0445	0.0827
35	0.2407	0.1865	0.1820	0.1024	0.0371	0.0689
40	0.1924	0.1491	0.1453	0.0817	0.0298	0.0551
45	0.1495	0.1161	0.1146	0.0648	0.0237	0.0437
50	0.1093	0.0851	0.0842	0.0477	0.0176	0.0322
55	0.0739	0.0578	0.0575	0.0327	0.0122	0.0222
60	0.0453	0.0355	0.0357	0.0204	0.0077	0.0139

**Table 3:** Radiometric calibration coefficients computed using proposed methodology for both RedEdge-3 and RedEdge-M sensors. The Blue, Green and Red bands produced very similar coefficients, while the NIR and RedEdge bands were different.

	Blue	Green	Red	NIR	RedEdge
**RedEdge-3**	3.656 × $10^{-8}$	2.916 × $10^{-8}$	5.863 × $10^{-8}$	5.168 × $10^{-8}$	5.396 × $10^{-8}$
**RedEdge-M**	3.233 × $10^{-8}$	2.814 × $10^{-8}$	5.698 × $10^{-8}$	3.726 × $10^{-8}$	6.524 × $10^{-8}$

**Table 4:** Average percent errors in radiance imagery for all bands using both conversion methods for the RedEdge-3. Standard deviations are shown in parenthesis.

Sensor	Method	Blue	Green	Red	RedEdge	NIR
RedEdge-3	MicaSense	−10.98 (0.93)	−0.43 (1.59)	3.59 (2.67)	32.81 (5.94)	−17.08 (2.45)
	Proposed (Average)	3.44 (5.63)	2.93 (5.47)	2.93 (6.63)	3.70 (8.91)	0.72 (4.08)
	Proposed (Closest)	3.66 (6.05)	3.19 (5.95)	3.25 (7.17)	4.23 (9.74)	0.75 (3.95)

**Table 5:** Average percent errors in radiance imagery for all bands using both conversion methods for the RedEdge-M. Standard deviations are shown in parenthesis.

Sensor	Method	Blue	Green	Red	RedEdge	NIR
RedEdge-M	MicaSense	−9.22 (0.61)	0.50 (1.70)	0.82 (1.79)	−1.62 (2.72)	1.58 (3.66)
	Proposed (Average)	2.50 (4.82)	2.91 (5.34)	1.28 (4.31)	0.99 (4.51)	0.78 (5.05)
	Proposed (Closest)	2.82 (5.41)	3.14 (5.78)	1.70 (5.01)	1.10 (4.55)	0.78 (4.86)

**Table 6:** Grass target reflectance factor errors as a result of calibration errors.

Sensor	Method	Blue	Green	Red	RedEdge	NIR	NDVI	NDRE
**SVC**	**Reference**	**0.022**	**0.071**	**0.026**	**0.188**	**0.490**	**0.899**	**0.445**
RedEdge-3	MicaSense Correction	0.020	0.070	0.027	0.249	0.406	0.876 (0.005)	0.239 (0.026)
	Proposed Correction	0.023	0.073	0.027	0.193	0.487	0.897 (0.007)	0.435 (0.038)
RedEdge-M	MicaSense Correction	0.020	0.071	0.026	0.185	0.491	0.899 (0.004)	0.458 (0.018)
	Proposed Correction	0.022	0.073	0.026	0.189	0.491	0.898 (0.006)	0.444 (0.027)

**Table 7:** RedEdge-3 12-bit digital count error for various light level, gain and exposure combinations. Digital count error was computed using vignette and row-corrected imagery from an integrating sphere. A positive correlation is seen between digital count error and both gain and exposure time, while a negative correlation is seen for digital count error and light level.

% Closed	Peak Radiance	Exposures	Gain	Blue	Green	Red	NIR	RedEdge
	[W/m${}^{2}$/sr/nm]	[ms]						
50	0.1093	0.585	1	49.664	51.681	25.783	18.749	22.480
50	0.1093	0.585	2	98.905	102.491	51.271	36.732	44.843
55	0.0739	0.765	1	41.940	44.367	22.605	36.732	44.843
55	0.0739	0.765	2	83.420	88.443	44.711	34.432	39.722
60	0.0453	0.585	1	29.103	30.208	16.466	14.697	15.587
60	0.0453	0.585	2	57.727	59.945	32.609	28.981	30.257
60	0.0453	0.585	4	114.67	119.22	64.870	56.984	60.723

**Table 8:** RedEdge-3 gain error for various light level, gain and exposure combinations. Digital count imagery was captured using an integrating sphere using a variety of light levels, gains and exposure times. Gain error was calculated by comparing the measured digital counts between images where the gain was the only variable difference.

% Closed	Peak Radiance	Exposures	Gain Difference	Blue	Green	Red	NIR	RedEdge
	[W/m${}^{2}$/sr/nm]	[ms]						
50	0.1093	0.585	1× to 2×	0.00022	0.00022	0.00027	0.00076	0.00043
55	0.0739	0.765	1× to 2×	0.00014	0.00015	0.00023	0.00068	0.00053
60	0.0453	0.585	1× to 2×	0.00025	0.00056	0.00078	0.00080	0.00121
60	0.0453	0.585	2× to 4×	0.00029	0.00040	0.00049	0.00058	0.00074
60	0.0453	0.585	1× to 4×	0.00055	0.00094	0.00140	0.00174	0.00262

**Table 9:** RedEdge-3 exposure time errors for various settings. Errors were computed using an oscilloscope and measuring the line produced by a moving dot. A positive correlation can be seen between exposure time and error.

Exposure Time [ms]	Error
0.5	2.7072 × $10^{-6}$
1.0	4.5643 × $10^{-6}$
2.5	1.0740 × $10^{-5}$

**Table 10:** RedEdge-3 radiometric calibration standard errors. Computed using the standard error for a regression estimate for all bands. Only 25 samples went into this computation, as the regression estimate itself contained only 25 samples.

Blue	Green	Red	NIR	RedEdge
3.988 × $10^{-10}$	3.268 × $10^{-10}$	8.118 × $10^{-10}$	4.841 × $10^{-10}$	1.062 × $10^{-9}$

**Table 11:** RedEdge-3 downwelling light sensor (DLS) standard errors. DLS measurements made using an integrating sphere at various light levels. Standard errors computed from 30 images at each light level. A positive correlation is seen between light level and the error.

% Closed	Peak Radiance	Blue	Green	Red	IR	RedEdge
	[W/m${}^{2}$/sr/nm]					
0 and 20	0.8023	3.6641 × $10^{-4}$	2.1970 × $10^{-4}$	1.1931 × $10^{-4}$	4.6659 × $10^{-5}$	8.8712 × $10^{-5}$
0 and 40	0.6331	1.2486 × $10^{-4}$	1.2858 × $10^{-4}$	1.0518 × $10^{-4}$	3.2586 × $10^{-5}$	7.9633 × $10^{-5}$
0 and 60	0.4989	2.9821 × $10^{-5}$	3.0761 × $10^{-5}$	3.8348 × $10^{-5}$	1.0106 × $10^{-5}$	2.3651 × $10^{-5}$
0 and 80	0.4581	3.8725 × $10^{-5}$	3.2880 × $10^{-5}$	2.9041 × $10^{-5}$	8.7316 × $10^{-6}$	2.7989 × $10^{-5}$
100 and 20	0.3585	7.2181 × $10^{-5}$	6.6139 × $10^{-5}$	5.0755 × $10^{-5}$	1.5182 × $10^{-5}$	3.7861 × $10^{-5}$
100 and 40	0.1906	4.1037 × $10^{-5}$	3.5980 × $10^{-5}$	2.7043 × $10^{-5}$	8.4237 × $10^{-6}$	2.2069 × $10^{-5}$
100 and 60	0.0449	1.0027 × $10^{-6}$	1.4695 × $10^{-5}$	6.4848 × $10^{-7}$	3.5597 × $10^{-6}$	1.0256 × $10^{-5}$
100 and 80	0.0017	5.8776 × $10^{-8}$	3.9563 × $10^{-8}$	4.0075 × $10^{-8}$	2.2207 × $10^{-8}$	2.7876 × $10^{-8}$

**Table 12:** Percentage error in radiance of the radiance image. IR and RedEdge produced higher radiance errors under the MicaSense methodology.

Method	Blue	Green	Red	IR	RedEdge
MicaSense	3.85	3.25	5.84	12.23	7.35
Proposed	3.75	3.21	5.45	9.29	6.54
